# Bioconjugation Strategies for Revealing the Roles of Glycerophospholipids in Cells

**DOI:** 10.1002/cbic.70428

**Published:** 2026-06-13

**Authors:** Brice Beauvais, Rajendra Rohokale, Sevin Mamputu, Ion Lupu, Zhongwu Guo, Sébastien Balieu

**Affiliations:** ^1^ INSA Rouen Normandie CNRS CARMeN UMR 6064 INC3M FR 3038 ENSICAEN University of Rouen Normandie, University of Caen Normandie, Normandie University Rouen France; ^2^ Department of Chemistry University of Florida Gainesville Florida USA

**Keywords:** bioconjugation, biological probes, bioorthogonal ligation, click chemistry, glycerophospholipids

## Abstract

Glycerophospholipids (GPLs) constitute one of the most important subclasses of phospholipids. These biomolecules are known to play multiple roles at the cellular level and are also involved as major constituents in membrane bilayers, cellular compartmentalization, and intramolecular signaling. To decipher their complex and diverse functions in cells, researchers have developed advanced bioconjugation strategies including chemical total synthesis of lipids from scratch, enabling the construction of precise lipid analogs. Bioorthogonal chemistry was also widely used to obtain lipid probes, such as fluorogenic and photoreactive GPLs. This interdisciplinary field of research merging, among other things, synthetic biology, live‐cell imaging, and bioorthogonal reactions provides unprecedented knowledge about lipid metabolism, transport, and interaction, thereby paving the way for functional studies and therapeutic innovations. Here, we summarize the approaches used to design glycerophospholipidic probes to explore lipidomics and various biological functions of GPLs.

## Graphical abstract

Glycerophospholipids are the main class of phospholipids, essential for membrane structure, compartmentalization, and cellular signaling. To study their roles, innovative probes and advanced bioconjugation strategies have been developed, including by de novo lipid synthesis and bioorthogonal conjugation chemistry. This interdisciplinary field combines biology and chemistry to explore lipid metabolism, dynamics, and interactions allowing deeper functional insights and therapeutic innovation.

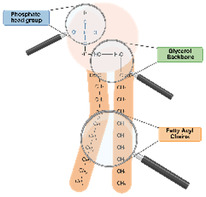

## 1 Introduction

Glycerophospholipids (GPLs) are biomolecules that play multiple roles at the cellular level and are also involved as major constituents in membrane bilayers [1], cellular compartmentalization [2], and intramolecular signaling [3]. GPLs are built around a glycerol backbone bearing up to two fatty acyl chains. The representative compositions of the fatty acyl chains include a saturated fatty acyl chain of varying lengths at the *sn‐*1 position of glycerol and a mono or polyunsaturated fatty acyl chain of varying lengths at the *sn‐*2 position. When one of these two positions is delipidated, it gives rise to lysoglycerophospholipids. These parameters add the first layer of complexity by allowing dozens of possible combinations on the hydrophobic part of GPLs. The second layer of complexity is afforded by the nature of the polar phosphate headgroup at the *sn‐*3 position. It can be diversely functionalized with moieties from the simplest phosphatidic acid (PA)—a phosphate monoester—to more sophisticated phosphate diesters, such as phosphatidylethanolamine (PE), phosphatidylcholine (PC), phosphatidylserine (PS), phosphatidylglycerol (PG), phosphatidylinositol (PI), phosphatidylinositol phosphates (PIPs), and glycophosphatidylinositols (GPIs). Even if not all possibilities are present in specific cells or particular compositions (fatty acyl chains and polar heads) are preferred for certain GPLs such as in the case of PIs (18:0‐20:4) [4], it remains that hundreds of compositions are still used by each organism as demonstrated by mass spectrometry analysis [5]. This fatty soup of GPLs in cells suggests that each lipid could potentially have nonoverlapping biological roles. Furthermore, given their rapid and complex metabolism [6] and intertwining with each other, establishing the roles of an individual lipid remains challenging. To circumvent this limitation, the use of probes with structures mimicking GPLs emerged during the 1970s [7, 8, 9] and has since undergone tremendous development [10]. This review will focus on the different bioconjugation strategies used to design probes for various families of GPLs. Essentially, there are three potential ways for GPL functionalization, including the modification of their polar phosphate headgroup, their fatty acyl chains, and the glycerol core, all of which will be covered in this review.

## 2 Bioconjugation Using the Fatty Acyl Chains

The bioconjugation of GPLs using their fatty acyl chains is a key strategy for developing lipidic probes, allowing the introduction of functional groups while globally preserving the amphiphilic nature of these biomolecules. However, the *sn‐*1 and *sn‐*2 chains are not treated in an equal manner. Indeed, the vast majority of modifications were carried out at the *sn‐*2 position, due to better structural tolerance and simplified synthesis. The fatty acyl chain at the *sn‐*2 position is generally unsaturated, exhibiting more tolerance to the introduction of bulky groups, whereas the *sn‐*1 chain is predominantly saturated, making its modification more disruptive to membrane organizations and far less biologically tolerated. In addition, the transesterification between the *sn‐*1 and *sn‐*2 positions in lyso‐intermediates adds another synthetical limitation. This side‐reaction restricts access to selectively modified analogs. Moreover, enzymatic specificity within biological systems, particularly that of phospholipases that preferentially target the *sn‐*2 position, has inexorably directed the development of probes toward this position. Consequently, modifying the *sn‐*1 fatty acyl chain for bioconjugation remains a secondary target, although it offers interesting prospects for modulating the biophysical properties of membranes.

In this section, we will focus on first the bioconjugation strategies using molecules having various chemobiological tools and a carboxylic acid group to functionalize the alcohol at the *sn‐*2 position and then the strategies using the metabolic pathway of the GPLs, to conclude with the specific bioconjugation at the *sn‐*1 fatty acyl chain.

### 2.1 Bioconjugation at the sn‐2 Position Using Synthetical Approaches

**FIGURE 1:**
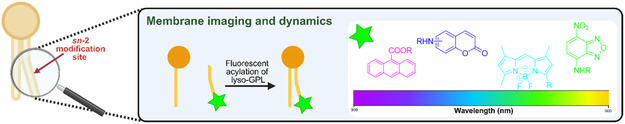
Synthesis of functionalized GPLs through acylation of lyso‐GPLs and the representative fluorophores used for the functionalization of GPLs at the *sn‐*2 position, ranging from aromatic fluorophores (anthracene, coumarin) to compact probes like NBD and BODIPY.

Modifying GPLs at their *sn‐*2 position is one of the most commonly used strategies for designing functional lipid probes. This preference stems from a unique combination of synthetic feasibility, structural tolerance, and biological relevance. From a synthetic perspective, the functionalization of the *sn‐*2 position relies primarily on the acylation of lyso‐GPLs (Figure 1)—a robust, regioselective transformation that has been well established since the earliest phospholipid syntheses [11, 12]. In particular, lyso‐GPLs at the *sn‐*1 position are thermodynamically and kinetically more stable than their *sn‐*2 counterparts, limiting acyl migration and allowing for the controlled introduction of modified chains at the *sn‐*2 position. Conversely, access to lyso derivatives at the *sn‐*2 position is more challenging due to their instability and tendency to rearrange to the *sn‐*1 form, which greatly complicates strategies for selective functionalization at the *sn‐*1 position.

From a biological perspective, this asymmetry is reinforced by the natural distribution of fatty acids within GPLs. The *sn‐*2 position is generally occupied by unsaturated lipid chains, conferring greater conformational flexibility and better tolerance to the introduction of chemical moieties, including bulky or functional groups. Conversely, the *sn‐*1 position is predominantly occupied by saturated fatty acids that play a key role in lipid packing and bilayer stability. Any modification at the *sn‐*1 position thus tends to more strongly disrupt the membrane organization and biophysical properties of the system. Furthermore, the *sn‐*2 position is the preferred site of action for many enzymes involved in lipid metabolism, particularly phospholipases A_2_ (PLA_2_), which specifically hydrolyze the *sn‐*2 ester bond. This enzymatic reactivity, although it may affect the stability of the probes, also offers a unique opportunity for the development of functional systems sensitive to enzymatic activity, particularly in approaches involving activatable probes or lipid metabolism monitoring.

Early approaches in this field involved either multistep syntheses of various carboxylic acid molecules bearing fluorescent moieties or use of natural polyene fatty acids, such as parinaric acid, which were among the first to be used as intrinsic fluorescent probes for studying membranes, particularly for analyzing phase transitions [13]. These systems were subsequently supplemented with aromatic fluorophores, such as anthracene derivatives [14], added to an oxidized fatty chain bearing a carboxylic acid function *via* diazo chemistry. The introduction of 7‐nitrobenz‐2‐oxa‐1,3‐diazole (NBD) fluorophore (Figure 1) by nucleophilic substitution between an amine and an alkyl halide marked a significant step toward more compact probes [15]. The work by Huster et al. [16] demonstrated that NBD‐labeled phospholipids could be incorporated into lipid bilayers and used to probe membrane dynamics. However, subsequent studies by Weng et al. [17] revealed significant disruptions induced by this fluorophore. This finding was already reported in 1990 by Chattopadhyay [18], who described the “snorkeling” effect, which corresponds to fluorophore relocations toward the membrane/water interface rather than to the hydrophobic core. These limitations highlighted a fundamental point, i.e., even minor modifications at the *sn‐*2 position can significantly alter the behavior of the lipid probe. The use of carboxylic acid BODIPY (Figure 1)—a more lipophilic fluorophore—primarily solved this drawback [19] but it requires a challenging multistep synthesis. In 2001, Farber, Pack, et al. [20] demonstrated the relevance of fluorescent‐PC BODIPY with in vivo FLIM‐FRET studies on Zebrafish. In line with this result, in 2013, Wang et al. [21] introduced the 7‐mercapto‐coumarin moiety via nucleophilic substitution to produce another type of fluorescent‐PC to perform FLIM‐FRET experiments over PLA_2_ enzymes as well.

**FIGURE 2:**
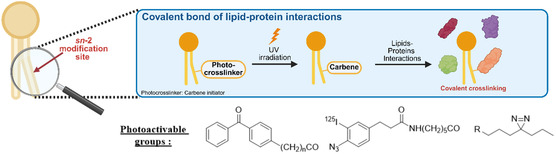
Phospholipids functionalized at the *sn‐*2 position with photoactivatable groups, including diazirine, benzophenone, and aryl azides. Upon UV irradiation, these moieties generate highly reactive intermediates (carbenes, diradicals, or nitrenes), enabling covalent cross‐linking with proximal membrane proteins.

Photoactivatable lipids, which typically require multistep syntheses, have emerged as powerful tools for studying lipid–protein interactions. The pioneering work of Gupta et al. [22] demonstrated that phospholipids containing carbene precursors, such as the diazirine moiety, could form covalent bonds with neighboring biomolecules following UV irradiation (Figure 2). This approach was subsequently extended to lipids containing benzophenone groups [23] via acyl chain functionalization, typically achieved by esterification of lyso‐PC to introduce the benzophenone moiety along the fatty acyl chain. Aromatic azides incorporated within lipid acyl chains were also used by Schroit et al. (Figure 2) [24] to investigate lipid–protein interactions in human erythrocyte membranes. Although examples of organometallic transformations (e.g., palladium‐catalyzed reactions) on membrane lipids remain relatively rare in biological contexts, this strategy is conceptually important since they expand the definition of lipid bioorthogonality beyond classical cycloadditions. In particular, they offer the possibility of selectively modifying pre‐existing double bonds, which are inherently compatible with lipid bilayers.

**FIGURE 3:**
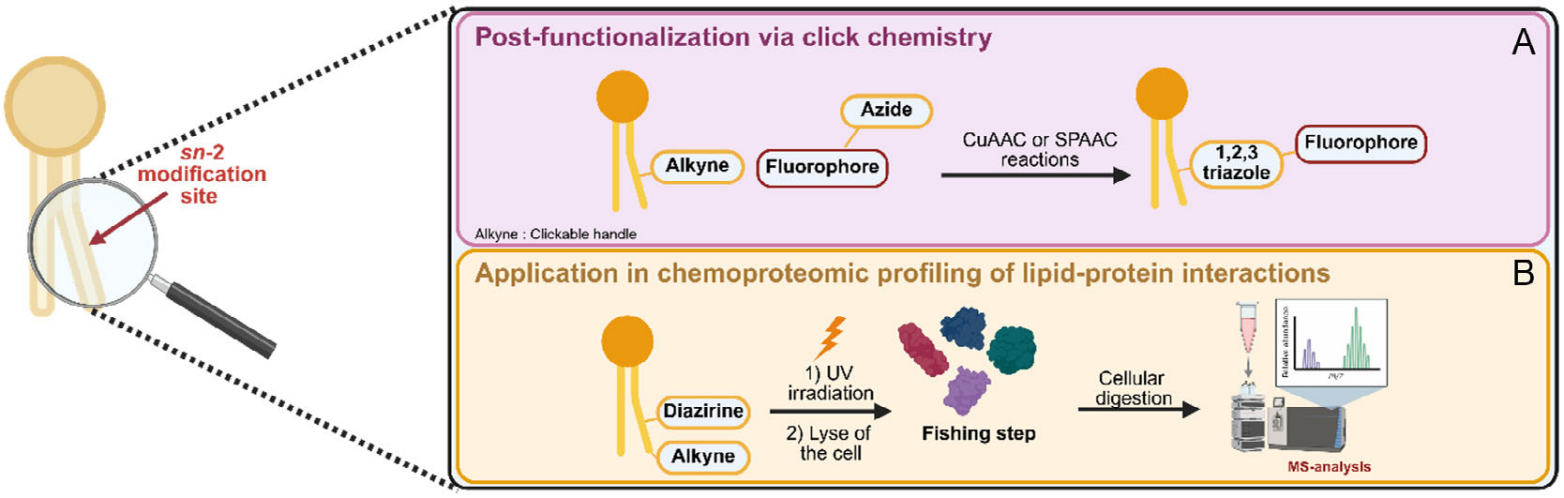
Bioorthogonal strategies for the functionalization of GPLs at the *sn‐*2 position. (A) Lipids bearing minimal bioorthogonal handles, such as alkynes or azides, can be incorporated into membranes with limited perturbation and subsequently derivatized via click reactions to introduce fluorophores or affinity tags. (B) Bifunctional lipid probes combining a photoactivatable group (e.g., diazirine) and a clickable moiety enable the covalent capture of lipid–protein interactions upon UV irradiation, followed by selective enrichment and identification through proteomic analysis.

Due to the demanding multistep syntheses for previous bioconjugate probes, azide and alkyne functional groups that can be easily introduced at various positions of the fatty acyl chain and are often commercially available have emerged as essential chemical tools for *sn‐*2 lipid modification (Figure 3). Their success stems from their ability to be easily integrated into fatty acid chains while preserving the physicochemical properties of lipids and membrane behavior and being readily functionalized by click chemistry such as CuAAC or SPAAC ligation (Figure 3) to afford new probes, thanks to their small size, chemical stability, and compatibility with bioorthogonal reactions [25]. Gaebler et al. [26] demonstrated that alkyne lipids can serve as substrates in enzymatic assays, confirming their compatibility with enzymes involved in lipid metabolism and highlighting their potential for incorporation at the *sn‐*2 position. At the same time, Ueshima et al. [27] have developed GPL precursors modified with azide groups, demonstrating that these groups can be introduced at the level of lipid biosynthesis and subsequently tracked using click chemistry. Beyond simple labeling, these motifs have also enabled the development of multifunctional lipid probes combining click chemistry and photoaffinity labeling. In this context, Kroon et al. [28] have developed phospholipids bearing both a photoactivatable diazirine or benzophenone group and clickable azide on the *sn‐*2 acyl chain, facilitating the covalent capture of lipid‐protein interactions followed by selective enrichment. This dual‐function design has since become a cornerstone of lipid interactomics. More recently, Korn et al. [29] systematically evaluated phospholipids modified with azide and diazirine, demonstrating that these minimal modifications at the *sn‐*2 position preserve membrane insertion while enabling efficient cross‐linking and downstream analysis. Collectively, these studies establish azide/alkyne chemistry as the gold standard for *sn‐*2‐bioorthogonal lipid engineering, combining ease of synthesis, biological compatibility, and analytical versatility (Figure 3).

### 2.2 Bioconjugation of the *sn*‐2 Lipid Chain Using the Metabolic Pathway

**FIGURE 4:**
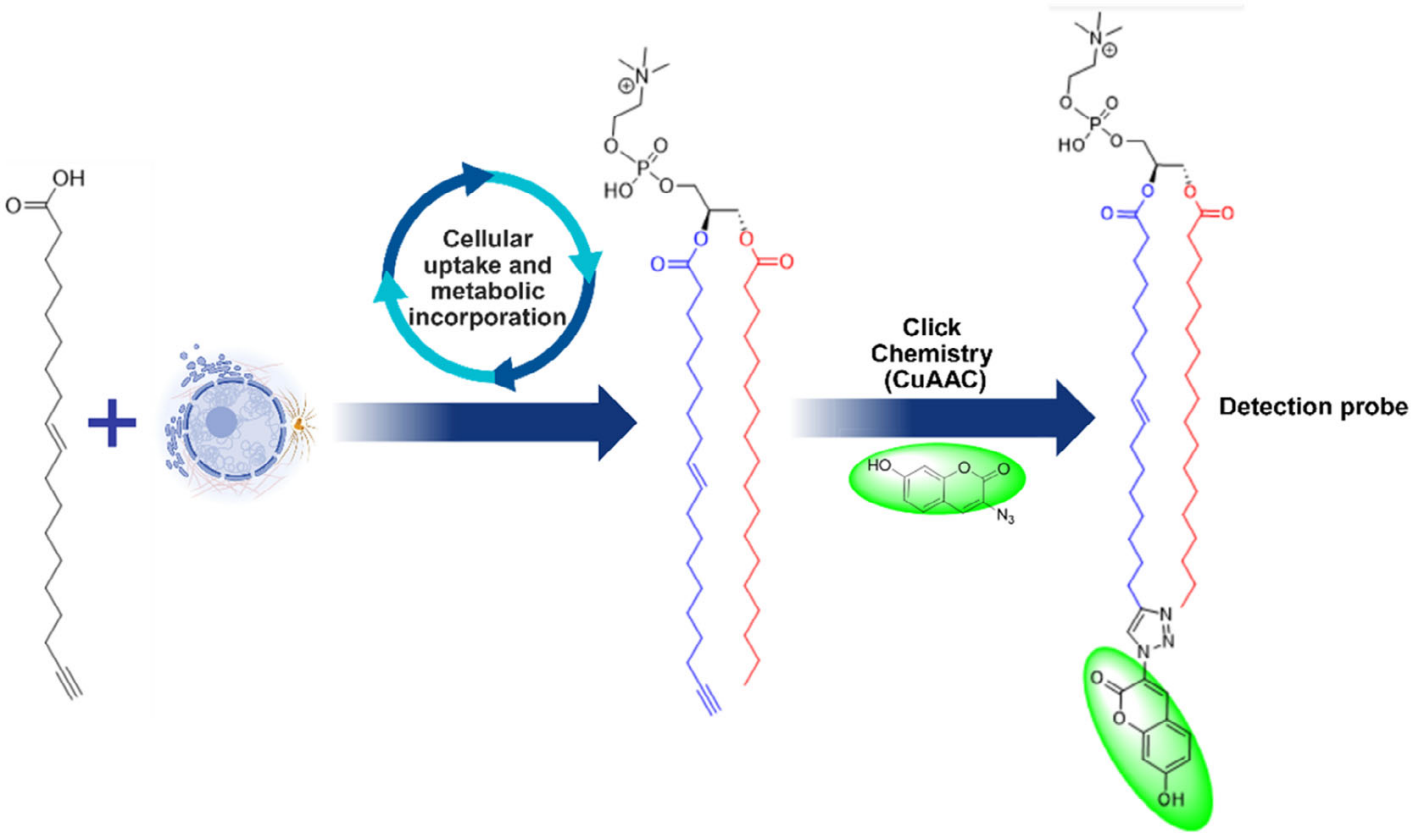
Metabolic incorporation of fatty acids bearing bioorthogonal azide or alkyne groups into phospholipids, predominantly at the *sn‐*2 position, and subsequent CuAAC reactions enable selective tagging and visualization of lipid metabolism.

The metabolic incorporation strategy for modified fatty acids relies on the ability of cells to take up and metabolize exogenous fatty acids bearing bioorthogonal functionalities (Figure 4). Fatty acids functionalized with either azides or alkynes have been developed as partners for click reactions. The low steric hindrance and similar dipole moment of azide to that of natural functional groups like hydroxy group allow the efficient incorporation into cell membranes, while providing access to a variety of biocompatible cycloaddition reactions. A major advantage of this approach lies in its ability to generate modified lipids that are similar to their natural counterparts in terms of composition and membrane behavior. Nevertheless, it is highly dependent on lipid remodeling pathways, which can lead to a redistribution of the modified chains among different lipid classes and thus reduce positional specificity.

Once internalized, the modified fatty acids are converted into acyl‐CoA derivatives and then incorporated into LPAs via endogenous acyltransferases, thereby positioning the chemical moiety at the *sn‐*2 position. Thus, the use of terminal alkynes as bioorthogonal functional groups represents one of the most significant developments in this field. In a seminal study, Thiele et al. [30] demonstrated that fatty acids bearing an alkyne could be incorporated into lipids in vivo and subsequently modified via a copper‐catalyzed azide/alkyne cycloaddition (CuAAC), enabling the visualization and tracking of lipid internalization and metabolism with high sensitivity (Figure 4). Various examples using this strategy are illustrated in Table 1.

**TABLE 1:** Various GPL probes bearing modification on fatty acyl chains.

Position	Probe	Type of modification	Headgroup	Key applications	Advantages	Limitations	References
***sn‐*2**	*sn‐*2‐Phospholipid prodrug analogs	Headgroup‐modified phospholipids	PC	Colon‐targeted drug delivery	Improved PK and lipid‐targeted delivery	Reduced membrane recognition via headgroup modification	[31]
***sn‐*2**	*sn‐*2‐Anthracene	Anthracene fluorophore (acyl chain)	PC	Quantification HPLC	Hight sensibility	Not biomimetic	[14]
***sn‐*2**	*sn‐*2‐NBD‐labeled phosphatidylcholine	NBD fluorophore (acyl chain, ωω or mid‐chain)	PC	Membrane dynamics, flip‐flop	Polarity‐sensitive fluorophore → reports interfacial environment	Preferential interfacial localization biases membrane depth reporting	[16, 17, 18]
***sn‐*2**	*sn‐*2‐BODIPY‐labeled phosphatidylcholine	BODIPY fluorophore (acyl chain)	PC	Imaging, PLA2 assays	high brightness, high photostability	Low polarity sensitivity → limited depth information	[19, 32]
***sn‐*2**	*sn‐*2‐Coumarin‐labeled phosphatidylcholine	Coumarin fluorophore (*sn‐*2)	PC	FRET, PLA2 kinetics	Suitable for donor–acceptor FRET systems	Requires precise probe design	[21]
***sn‐*2**	*sn‐*2‐Quenched BODIPY phosphatidylcholine	Self‐quenched fluorophore (*sn‐*2)	PC	Fluorogenic PLA2 assays	Turn‐on signal upon hydrolysis → high sensitivity	Enzyme‐specific readout	[33]
***sn‐*2**	*sn‐*2‐Diazirine‐functionalized phosphatidylcholine	Diazirine (mid‐chain)	PC	Lipid–protein cross‐linking	Short‐lived carbene → high spatial resolution	Low cross‐linking yield, UV activation required	[29, 34]
***sn‐*2**	*sn‐*2‐Benzophenone‐modified phosphatidylcholine	Benzophenone (*sn‐*2)	PC	Interaction mapping	Long‐lived triplet state → efficient capture	Lower spatial specificity	[23]
***sn‐*2**	*sn‐*2‐Azido‐functionalized phosphatidylcholine	Azide (ω‐position)	PC	Click chemistry, metabolic tracing	Bioorthogonal, compatible with live cells	Requires secondary ligation chemistry	[27, 35]
***sn‐*2**	*sn‐*2‐Alkyne‐functionalized phosphatidylcholine	Terminal alkyne (ω‐position)	PC	Lipidomics, enrichment	Efficient click‐based enrichment	May alter local hydrophobicity	[36, 37]
***sn‐*2**	*sn‐*2‐Photocleavable phosphatidylcholine	Photolabile linker (*sn‐*2)	PC	Controlled release systems	Spatiotemporal control	UV‐induced phototoxicity	[38]
***sn‐*2**	*sn‐*2‐Fluorescent‐ phosphatidylcholine	Fluorophore (*sn‐*2)	PC	Membrane organization	Little disruption of the membrane	Lower signal intensity	[39]
***sn‐*1**	*sn‐*1‐Azido‐functionalized phosphatidylcholine	Azide (*sn‐*1)	PC	Cellular imaging	Compatible with in cellulo click chemistry	May interfere with lipid transport	[25]
***sn‐*1**	*sn*‐1‐Radiolabeled phosphatidylcholine	Isotopic label (*sn‐*1 acyl chain)	PC	Lipid transfer studies	High quantitative sensitivity	No spatial imaging capability	[24]
***sn‐*1/*sn‐*2**	*s* *n*‐1,2‐Bis‐NBD‐labeled phosphatidylcholine	Dual NBD fluorophores	PC	Lateral organization	Signal amplification	Strong membrane perturbation	[40]
***sn‐*1/*sn‐*2**	*sn*‐1,2‐Bis‐BODIPY phosphatidylcholine	Dual BODIPY	PC	Lo/Ld phase separation	High brightness	Steric bulk alters membrane properties	[41]
***sn‐*1/*sn‐*2**	*sn*‐1,2‐FRET‐pair phosphatidylcholine	Donor–acceptor fluorophores	PC	Enzymatic kinetics	Direct distance measurements	Complex synthesis	[21]
***sn‐*1/*sn‐*2**	*sn‐*1,2‐Bis‐diazirine phosphatidylcholine	Dual diazirine groups	PC	Interaction networks	Multipartner capture	Increased nonspecific cross‐linking	[22]
***sn‐*1/*sn‐*2**	*sn‐*1*‐*Azido/sn‐2‐diazirine phosphatidylcholine	Click + photo‐cross‐linker	PC	Protein identification	Combined capture and enrichment	Multistep protocols	[28, 42]
***sn‐*1/*sn‐*2**	*sn‐*1,2*‐*Bis‐alkyne phosphatidylcholine	Dual alkyne groups	PC	Advanced lipidomics	Dual labeling sites	Increased membrane perturbation	[37]
***sn‐*1/*sn‐*2**	Trifunctional phosphatidylserine probe	Fluorophore + photo + click	PS	Lipid–protein interactome	Multimodal capability	Synthetic complexity	[43]
***sn‐*1/*sn‐*2**	Caged phosphatidylcholine analog	Photocaging group (*sn‐*2)	PC	Controlled activation	Precise spatiotemporal control	UV requirement	[44]
***sn‐*1/*sn‐*2**	Fluorogenic phospholipase reporter lipids	Fluorogenic substrate	PC / PI	PLA2/PLC activity assays	High sensitivity	Enzyme‐specific applicability	[33, 45]
***sn‐*1/*sn‐*2**	Modified phosphatidylinositol (PI(4,5)P2) probes	Headgroup + acyl modifications	PI	Lipid signaling	High biological specificity	May alternative recognition	[46]
***sn‐*1/*sn‐*2**	Multifunctional phosphoinositide probes	Multiple functional groups	PI	Imaging + biochemistry	Highly informative	High synthetic complexity	[47]
***sn‐*1/*sn‐*2**	Ether lipid bifunctional probes	Modified ether‐linked chains	Ester vs. ether lipids	Intracellular transport	Physiological relevance	Synthetic difficulty	[48]
***sn‐*2/*sn‐*1**	PLA2‐specific glyceric acid phospholipids	Glyceric acid scaffold replaces glycerol backbone	PC	PLA2 substrate specificity assays	Scaffold‐engineered enzymatic selectivity	Non‐native backbone reduces physiological relevance	[49]

### 2.3 Bioconjugation of the *sn*‐1 Position

Compared to the extensively explored *sn‐*2 position and headgroup modifications, the *sn‐*1 fatty acyl chain of GPLs remains comparatively underutilized for bioconjugations. This is primarily due to its structural and functional role in membrane architecture, where the *sn‐*1 chain is typically saturated and contributes significantly to lipid packing and bilayer stability. Therefore, chemical perturbations at this position are more likely to induce alterations in membrane organization, imposing strict constraints on probe design. However, some studies have indirectly demonstrated that the *sn‐*1 position can be exploited for the introduction of functional groups, showing that an appropriate balance can be maintained between their hydrophobicity and membrane compatibility. In this context, Waybright et al. [50] have shown that the enzymatic activity of phospholipases depends heavily on the hydrophobicity of the acyl chains and the substituents incorporated into them, suggesting that modifications at the *sn‐*1 position may be tolerated if they do not excessively disrupt the lipid environment. In addition, the intrinsic instability of lysophospholipids having acyl chain at *sn‐*2 position and the propensity for acyl migration from *sn‐*2 position to *sn‐*1 further complicate the selective and stable functionalization of this site involving protection‐deprotections steps during their synthesis. Nevertheless, a number of studies have demonstrated that the *sn‐*1 position can be chemically addressed in a controlled manner to generate functional lipid probes.

Early strategies relied on stepwise glycerol scaffold functionalization to achieve regioselective access to the *sn‐*1 hydroxy group, enabling subsequent coupling with fatty acyl chains or reporter groups. These approaches were applied to phosphatidic acid and related lipid species for affinity‐based proteomic studies. For example, lipid–affinity matrices derived from *sn‐*1‐functionalized phospholipids enabled the selective capture of PA‐binding proteins, leading to the identification of key regulators of membrane trafficking, including coatomer complexes and small GTPases such as Arf [51, 52].

A major conceptual advance in *sn‐*1 lipid engineering emerged during the development of functionalized PI probes. In 2010, Holmes et al. [46] reported the synthesis of a complete family of amine‐functionalized PIP analogs, providing a flexible platform for PIP conjugation and biological evaluation. Importantly, this study demonstrated that phosphate‐proximal modifications can be introduced without abolishing molecular recognition or biological activity, thereby validating the tolerance of complex signaling lipids toward strategic derivatization near the *sn‐*1 region. This work opened the way to modular conjugation strategies for phosphoinositides and reinforced the idea that carefully positioned polar handles can preserve biological function while enabling chemical accessibility. In addition, Huang et al. [45] studied PI fluorescent substrates and demonstrated that the position and nature of the modifications could directly influence enzymatic recognition, suggesting the possibility of precise modulation through alternative substitutions at the *sn‐*1 position. In summary, the strategies presented by Rasmussen and Hermetter [40] have made it possible to access a wide variety of functionalized GPLs, including analogs in which the modification can be introduced on either acyl chain.

These approaches have led to multifunctional lipid probes, such as those reported by Bandyopadhyay and Bong [43], which combine photoactivatable groups, bioorthogonal functions, and detection modules within a single molecule. In particular, they have enabled the design of lipids with simultaneous modifications of both acyl chains. Notably, a PI probe bearing a modification at the *sn‐*1 position was found to be capable of specifically interacting with the PT‐1 protein, thereby illustrating the functional potential of this position, which has otherwise been underutilized. Although this work is not exclusively focused on *sn‐*1 modification, it nevertheless demonstrates that this approach is compatible with advanced proteomics, provided that the impact of substituents on the overall properties of the lipid is rigorously controlled.

Beyond affinity‐based and headgroup‐adjacent modifications, several studies have also explored functionalization of the *sn‐*1 acyl chain using bioorthogonal and photoactivatable chemistry. Compared to *sn‐*2‐focused systems, these approaches remain less common but provide complementary information on lipid topology and membrane interactions. Azide‐ and alkyne‐bearing lipid analogs have been introduced as minimal bioorthogonal handles compatible with cellular environments and subsequent click chemistry labeling. In parallel, photoactivatable probes incorporating benzophenone or diazirine groups have been employed to capture transient lipid–protein interactions in membranes, enabling covalent cross‐linking upon UV irradiation followed by proteomic identification of interacting partners [53]. In addition, enzymatic usage has further illustrated the application of *sn‐*1‐modified lipid probes. Alkyne‐functionalized lysophospholipids have been used to quantify lysophosphatidic acid acyltransferase activity and to determine kinetic parameters such as *K*_m_ and *V*_max_, demonstrating that minimal modifications at or near the *sn‐*1 position can be compatible with enzymatic recognition [36, 37]. Furthermore, activity‐based lipid probes have also been developed to target phospholipase subclasses in proteomic environments, reinforcing the applicability of *sn‐*1‐compatible chemical handles for functional enzymology studies [54]. Collectively, these studies demonstrate that although the *sn‐*1 position is more synthetically and biologically constrained than *sn‐*2, it can nevertheless serve as a functional site for lipid engineering when modifications are carefully designed. The combination of affinity‐based scaffolds, bioorthogonal handles, and photoactivatable chemistries now enables the development of increasingly sophisticated *sn‐*1 lipid probes, extending the scope of GPL chemistry toward more integrative chemoproteomic and cell biological applications.

## 3 Bioconjugation Using the Polar Phosphate Headgroup

Bioconjugation of GPLs through their headgroups must be considered carefully regarding their biological activities. Thus, these derivatives are typically unrelated to naturally occurring membrane lipids in respect to charge, polarity and shape of the headgroup. Since most aspects of the behaviors of membrane lipids are headgroup‐dependent, they cannot be fully considered as lipid‐specific probes. This is particularly true for small heads like ethanolamine. Nevertheless, a relatively modest change in the headgroup structure can result in an important change in biological functions. Consequently, developing synthetic strategies generating bioconjugate GPLs *via* the headgroups that mimic their natural counterparts is challenging and, if possible, should be avoided.

### 3.1 Bioconjugation Using the Amine Function of PE, PS, or PC

**FIGURE 5:**
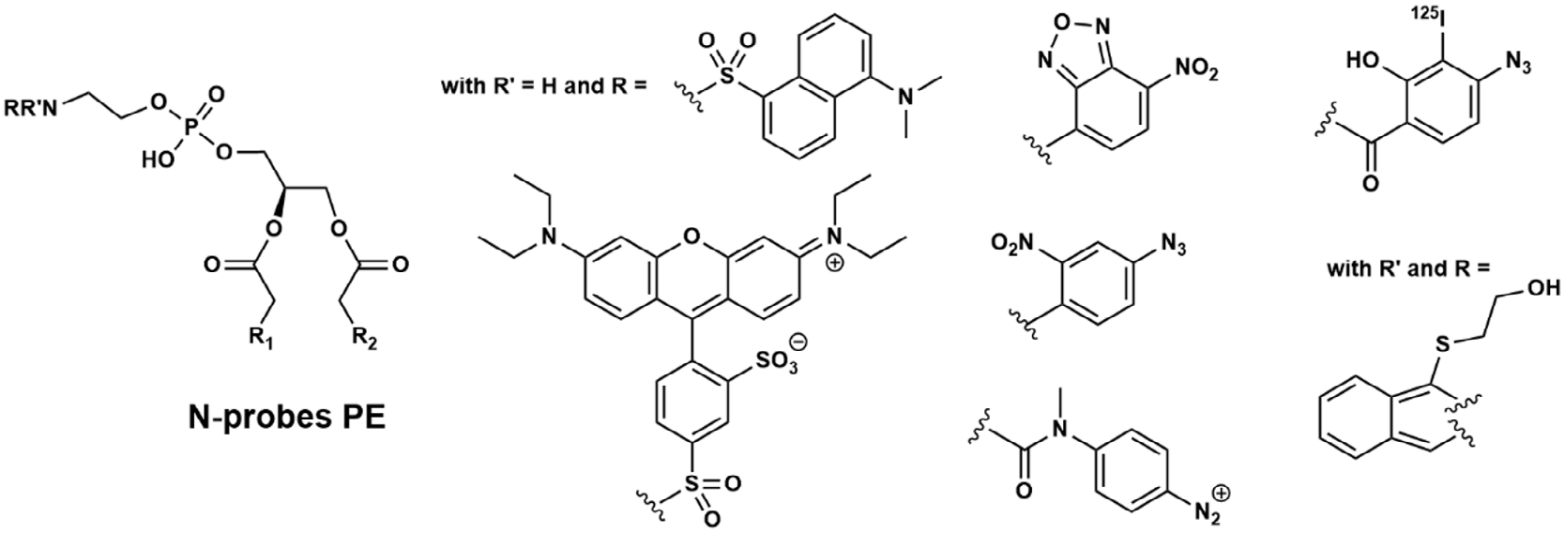
Various N‐modified PE probes containing dansyl, NBD, 4‐azido‐3‐[^125^I]iodo‐2hydoxybenzoyl, rhodamine, 4‐azido‐3‐nitrophenylaminoethyl, (1‐methylureido)benzenediazonium, and 1‐(2′‐thio‐1′‐hydroxyethyl)‐2‐isoindole fluorophores.

The development of fluorescent GPL probes by using the polar phosphate headgroup emerged with PE in the early 1970s, thanks to its presence of a nucleophilic primary amine. This strategy has evolved significantly over the past decades with paralleling advances in fluorescence spectroscopy and membrane biology. The first example is Waggoner and Stryer's work [55], where dansyl fluorophore (Figure 5) was covalently introduced via nucleophilic substitution, enabling the first fluorescence‐based study of biological membranes using fluorescent GPL. Throughout the 1970s, this probe was employed to study membrane polarity, fluidity, and lipid organization [56]. A major milestone was reached in the 1978 by Shaw et al. [57] with the introduction of NBD to label PE, which rapidly became a widely used strategy, due to its relatively small size and suitable photophysical properties that minimized perturbation of membrane structure as compared to dansyl. Then, various fluorescent [58, 59] and photoreactive [7, 60, 61] molecules were introduced following the same strategy (Figure 5).

**FIGURE 6:**
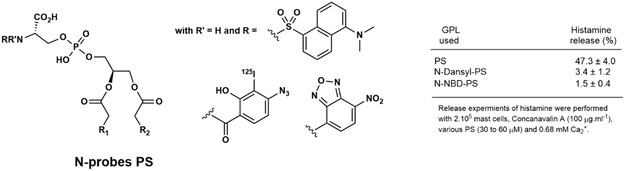
N‐Modified PS probes containing dansyl, NBD, and 4‐azido‐3‐[^125^I]iodo‐2hydoxybenzoyl fluorophores used to detect the histamine release as a mean percentage of total cell histamine in the supernatants of cell pellets.

The primary amine of PS was also used to perform bioconjugation for these biomolecules. Early efforts focused on the development of fluorescent PS probes. Fluorescent PS analogs were first developed by Harris and Stahl [62] and followed by Martin and Lagunoff [63] during the 1970s. The work of Martin and Lagunoff on the mast cell histamine secretion shows clearly that N‐modified PS (e.g., N‐dansyl‐PS and N‐NBD‐PS, Figure 6) are not able to reproduce the biological effect of native PS. Fluorescent PSs are even considered as inhibitors on mast cell secretion. In the early 1990s, Blanton and Wang [64] developed photoactivatable phospholipids, such as aryl‐azide derivatives of PS (Figure 6), in which photoreactive groups like azidosalicylic acid already applied to N‐modified PE probes were attached to the polar headgroup. Upon UV irradiation, these probes cross‐react with adjacent proteins to identify lipid–protein interactions at membrane interfaces including for example studies of nicotinic acetylcholine receptor [65].

PCs were the last amine‐bearing GPLs explored for bioconjugation through the headgroup since their amine exists in ammonium form, which does not have any nucleophilic property, making the previous strategies developed for PE and PS not applicable here. In 2009, de Kroon et al. [28] synthesized various N‐PhotoCrossLinker‐PC (N‐PCL‐PC) via monodemethylation of *sn‐*glycerophosphocholine utilizing 1,4‐diazabicyclo [2]octane (DABCO) and then reaction of the resulting tertiary amine with varied PCLs having benzylic bromide functionality. The probes, together with mass spectrometry analysis of cross‐linked proteins, were used to identify both established and potential new PC‐interacting proteins. In the same year, Cairo et al. [66] converted diacylglycerol into clickable N‐propargyl‐PC by exploiting the reactivity of 2‐chloro‐2‐oxo‐1,2,3‐dioxophospholane with alcohol. The resulting cyclic phosphate triester can be opened with N, N‐dimethylazidoethylamine to afford N‐propargyl‐PC, which is able to react with various azide species [67] by click chemistry to allow bioconjugation. Nevertheless, the bioconjugation of the headgroup involving multiple synthetic steps, including difficult purification processes, particularly in the case of PC, has taken a back seat in recent years in favor of an approach based on metabolic engineering (see Section 3.3).

### 3.2 Bioconjugation Using the Hydroxyl or Phosphoryl Groups of PI, PIP, and GPI

PI, PIPs, and GPIs constitute important subclasses of biosynthetically related membrane lipids, which play central roles in cellular organization and signaling [68]. Distinct phosphorylation patterns of PI on the *myo*‐inositol headgroup can generate molecular signals that regulate membrane trafficking, cytoskeletal dynamics, signal transduction, and organelle identity, whereas GPI anchors further exploit PI biochemistry to control the localization and functions of surface proteins [69]. Despite their importance, mechanistic studies on PI‐, PIP‐, and GPI‐mediated processes are hindered by their close structural similarity. To differentiate them, chemical synthesis and bioconjugation methods have become crucial platforms. In this context, both the lipid tails and polar headgroups of PI, PIPs, and GPIs have been used for the functionalization. Since lipid tail modifications were already discussed in the previous section, this section is mainly focused on the selective functionalization of their headgroups. This method is not likely to influence the properties of lipids, thereby preserving the organizations of PI, PIs, and GPIs in and interaction with the cell membrane. For the synthesis of these probes, different protection and deprotection tactics, functionalization strategies, and phosphorylation methods have been developed, which have greatly expanded access to various molecular probes, such as those containing fluorescent and affinity tags, photo‐cross‐linkers, or immobilized ligands, enabling diverse biological studies [10]. As a result, headgroup bioconjugation is accepted as a powerful framework for dissecting the functions of PI, PIPs, and GPI‐anchored proteins (GPI‐APs) and related biology [70, 71, 72, 73].

PI and PIP probes are usually derived from inositol and its phosphates (InsPs), which have rather limited functional diversity, primarily phosphoryl and hydroxy groups, constraining the sites and methods available for tagging. Thus, an earlier strategy involved replacing phosphoryl groups with phosphoesters containing a free amino group, which made further derivatization easier [74, 75]. For example, Prestwich et al. demonstrated the importance of such PIP probes for protein discovery and functional elucidation, helping define the concept that modified PIPs can serve not only as ligands but also as affinity reagents [76]. Although these designs prove to be useful for affinity capture, headgroup studies, and comparative profiling of PIP‐binding proteins, the tolerance for phosphate modification or extension remains context‐dependent.

**FIGURE 7:**
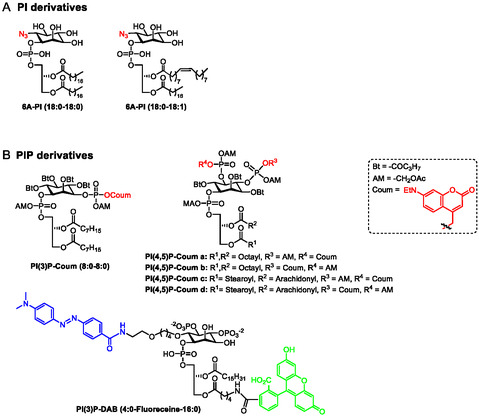
Structures of (A) PI‐probes **6A‐PI** (18:0‐18:0) and **6A‐PI** (18:0‐18:1), and (B) PIP‐probes **PI(3)P‐Coum** (8:0‐8:0), **PI(4,5)P‐Coum a-d**, and **PI(3)P‐DAB** (4:0‐Fluoresceine‐16:0).

The Guo group has developed several PI probes bearing various fatty acyl chains, such as **6A‐PI** (18:0‐18:0) and **6A‐PI** (18:0‐18:1) (Figure 7) [77], which aimed to selectively target PI and PIPs. These probes contain the PI moiety, which is expected to help their partitions into and pass‐through cell membranes, thereby improving their efficiency. Another useful feature of these probes is that the inositol 6‐hydroxy group is replaced with an azido group, blocking their participation in GPI biosynthesis to achieve selective PI and PIP targeting. Therefore, these probes constitute useful platforms for PI and PIP‐specific engineering and related investigations, as well as for the exploration of the influences of the PI lipid structure on its biological properties. It is also possible to use the nucleophilic character of the phosphate groups. For example, Schultz et al. [78] developed a membrane‐permeant, photoactivatable PI(3)P probe **PI(3)P‐Coum** (8:0‐8:0) (Figure 7B), enabling acute intracellular releases of this lipid to show that a localized increase of **PI(3)P‐Coum** (8:0‐8:0) is sufficient to drive EEA1‐dependent early endosomal fusion, providing direct in vivo evidence for the causal signaling role of PI(3)P. This strategy was successfully extended to PI(4,5)P_2_ probes **PI(4,5)P‐Coum a–d** (Figure 7B), capable of uncaging in cells within sub‐seconds to rapidly release PI(4,5)P_2_ and potentiate exocytosis, displaying synaptotagmin‐1 and Munc13‐2 as key downstream effectors [79]. Wang and co‐workers [45] introduced **PI(3)P‐DAB** (4:0‐Fluoresceine‐16:0) (Figure 7B) as a membrane‐associated fluorogenic PIP_2_ reporter, which has a fluorophore in the lipid and a DABCYL quencher at the inositol 6‐position. This probe can be selectively hydrolyzed by multiple mammalian PLC isozymes, but not by other PIP_2_‐metabolizing enzymes like PLD, PLA2, or PI3K. When it is cleaved, fluorescence increases; therefore, it has been utilized as a nonradioactive, functional surrogate for traditional membrane‐based PLC assays to capture membrane‐dependent PLC regulation, including Gαq‐mediated PLC‐β3 activation. These studies establish the 6‐OH position of PIP_2_ as a privileged site for conjugation to construct fluorescent and fluorogenic probes suitable for real‐time study of PIP_2_ and analysis of PLC activity.

**FIGURE 8:**
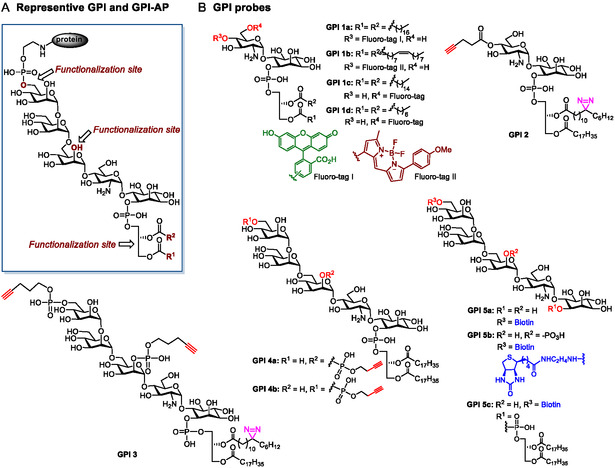
(A) Structures of a representative GPI‐AP carrying the conserved GPI core structure, and (B) functionalized synthetic GPI analogs or derivatives as GPI probes.

Despite the ubiquity of GPI‐APs (Figure 8A) in eukaryotic cells, their isolation and preparation remain challenging, due to their highly complex and heterogeneous structures, amphiphilic properties, post‐translational nature, and low abundance. Consequently, synthetic GPIs and GPI precursors useful for their metabolic engineering have become valuable tools for the investigation of GPIs and GPI‐APs. The most common functional groups used to modify GPIs and GPI precursors include azides and alkynes due to the reasons already discussed above. Although some of the inositol and PI probes with free 2,6‐hydroxy groups are useful for metabolic engineering of GPIs and GPI‐APs (see Section 3.3), their efficiencies can be influenced by their potential involvement in PIP pathways. Therefore, more selective and effective probes have been developed for GPI anchors and GPI‐APs. Such probes include both modified biosynthetic precursors for GPIs and functionalized GPIs, as shown in Figure 8B. They usually contain the intact conserved core structure or are short GPI analogs with sites involved in PIP biosynthesis blocked so that they can be GPI‐selective. Moreover, some of these GPI probes can be utilized to readily access GPI‐protein conjugates as GPI‐AP analogs or probes.

Fluorescently tagged GPI analogs **GPI 1a‐d** (Figure 8B) containing the core pseudodisaccharide and different lipids of varied saturation patterns and chain lengths were prepared to explore the influence of lipid structures on the behaviors of GPIs in membranes [80]. Studies using **GPI 1a‐d**, lipid‐remodeling mutants, PS‐depleted cells, and molecular simulations discover that GPIs with long saturated lipid chains have a higher tendency to create locally liquid‐ordered nanodomains and that GPI‐AP nanoclustering in the cell membrane is not only driven by GPI anchor but also governed by transbilayer coupling between their long, saturated lipid chains in the outer leaflet and that of PS in the inner leaflet, together with cholesterol and cortical actin, thus providing a general mechanistic framework for the membrane organizations of GPIs and related signaling platforms in cells [81].

In 2011, the Guo group established a versatile synthetic strategy for clickable GPI anchors carrying an alkyne or azide handle, which were efficiently coupled with imaging or affinity tags, thereby opening a route to functionalize GPIs for biological studies [82]. Doubly functionalized GPI analog **GPI 2** and GPI derivative **GPI 3** containing the core pseudodisaccharide and intact conserved core structure, respectively, having a photoreactive diazirine group attached to their lipid and clickable alkyne linked to their glycan were then designed and synthesized to examine GPI‐interactomes [83, 84, 85]. These probes were employed to capture GPI‐interacting proteins in live cells through UV‐promoted cross‐linking and click reaction‐based conjugation with an affinity tag to facilitate the enrichment of the cross‐linked proteins for proteomic analysis, leading to the identification of many known and new GPI‐associated membrane proteins. Comparing the results obtained with **GPI 2** and **GPI 3** further reveals that the glycan structure of GPIs determines the property of interaction and the proteins GPIs interact with. Moreover, a diversity‐oriented synthetic strategy was established to access many other GPI probes [86], such as **GPI 4a** and **GPI 4b** that have the alkynyl handle at different positions, which would facilitate various biological studies of GPIs.

Another type of GPI probe is designed to carry a biotin tag, which enables their conjugation with proteins based on the robust, strong binding between biotin and avidin/streptavidin. These probes are not only useful tools for various bioanalyses but also powerful, universal platforms for convenient, flexible, and rapid access to GPI‐AP analogs that are extremely difficult to come by [87]. For example, biotin‐labeled GPI core glycans **GPI 5a** and **GPI 5b**, which enable streptavidin–horseradish peroxidase conjugate‐based enzyme‐linked immunosorbent assay (ELISA), were utilized to evaluate the binding affinities between GPIs and pore‐forming toxin–CAMP factor, revealing their strong interactions and the significant influence of the inositol 1‐*O*‐phosphate group on these interactions [88]. On the other hand, biotin‐labeled **GPI 5c** has been shown to readily couple with avidinated recombinant proteins to provide various natural GPI‐AP‐mimetic GPI‐protein conjugates [89, 90], which are useful for the study of GPI‐APs. In another study, Suzuki, Komura, Kusumi, Kiso, and co‐workers have used a fluorescently labeled GPI‐AP, the human CD59, and single‐fluorescent‐molecule imaging in live‐cell membrane to reveal its clear but transient colocalization and codiffusion with gangliosides in cholesterol‐ and GPI‐dependent manners [91, 92].

Additionally, Silva and co‐workers prepared a series of GPI probes containing different glycans, lipids, and a free or acetylated amino group within the glucosamine residue (GlcNAc vs. GlcN) [93]. Biological studies using these probes show that synthetic GPI intermediates can rescue early, cytoplasmic steps of GPI biosynthesis in PIGA‐ and PIGL‐deficient HEK293 cells, while more elaborated intermediates designed to target ER luminal steps fail to restore biosynthesis efficiently, highlighting intracellular transport as a major and important step for therapeutic rescue strategies. Further studies by the same groups using simplified GPI fragments in monolayer membranes demonstrate that deacetylation is crucial for hydrogen‐bond‐driven head‐group organization, and lipid composition and unsaturation strongly influence packing, fluidity, and microdomain formation [94, 95].

The above examples and studies have demonstrated the general strategy for metabolic engineering of PI, PIPs, and GPIs using tagged biosynthetic precursors and the design of PI, PIP, and GPI probes through functionalization of their head groups. The resultant probes maintain the properties of the parent or target molecules, including their bioavailability and bioactivity. As a result, these probes with detectable reporters, which enable fluorescent imaging, affinity enrichment, and other analytical methods, have been widely used for various studies to gain more biological insight. Such studies have greatly advanced our knowledge and understanding of PI, PIPs, and GPIs, as well as their biomedical applications.

### 3.3 Bioconjugation of the Headgroup by Using the Metabolic Pathway of the GPL

An alternative to fully synthetic probe‐based GPL bioconjugations on the headgroup is manipulating their metabolic pathways [35] with bioorthogonally tagged biosynthetic precursors, such as propargylcholine (ProCho) or N‐_L_‐SerN_3_, in cell studies. This approach ensures that lipid analogs are produced with spatial and temporal fidelity comparable to their native counterparts. Moreover, the minimal size of the bioorthogonal functionality allows the resulting lipids to more closely match the natural species they are designed to mimic until to be converted into desired probes.

**FIGURE 9:**
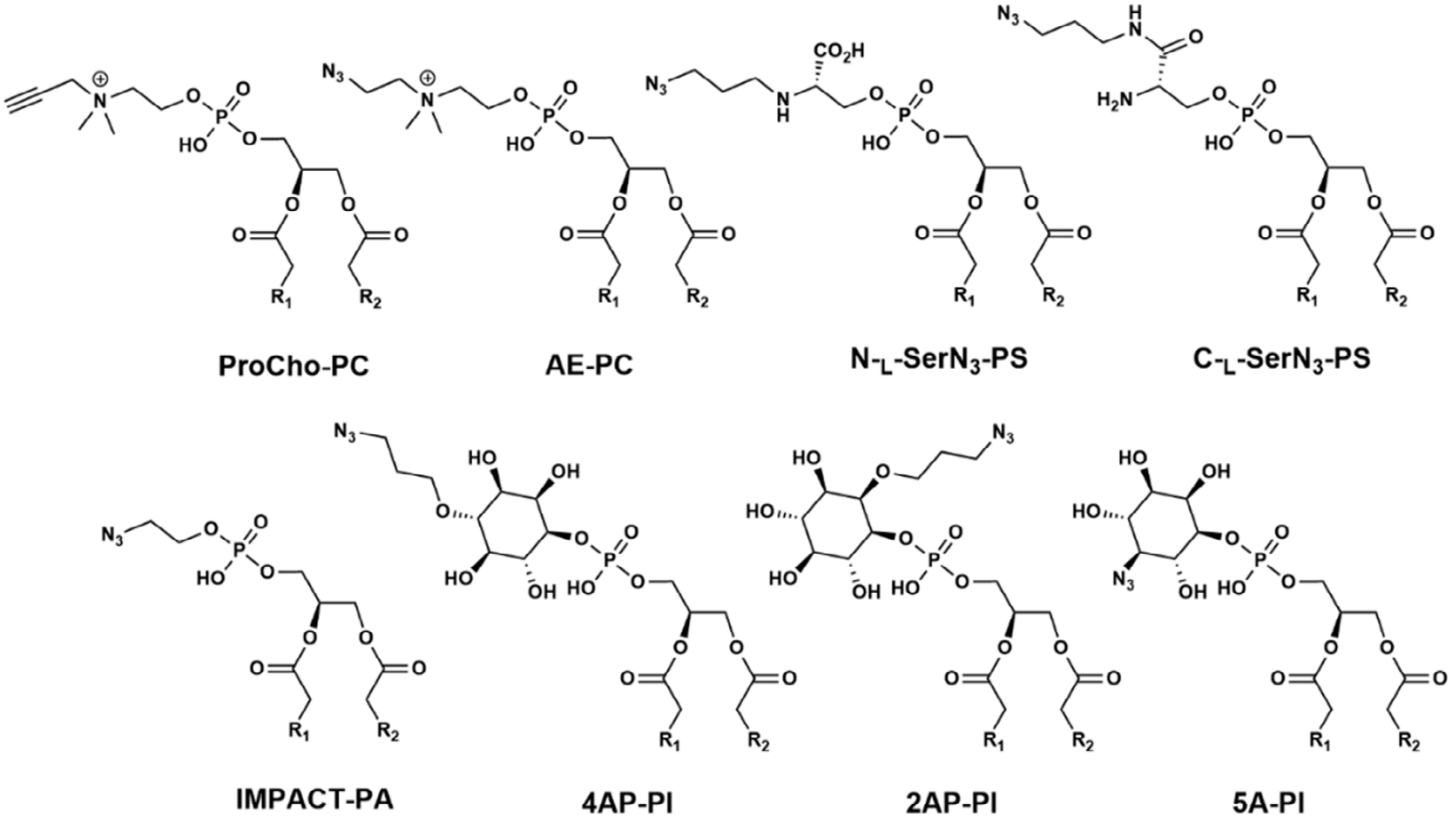
Various GPL probes obtained through engineering of metabolic pathways, including **ProCho‐PC**, **AE‐PC**, **N‐** **L** **‐SerN**_**3**_ **‐PS**, **C‐** **L** **‐SerN**_**3**_ **‐PS**, **IMPACT‐PA**, **2AP‐PI**, **4AP‐PI**, and **5A‐PI**.

In 2009, Salic et al. [96] used ProCho as a substrate for cellular incorporation into the Kennedy pathway, giving the cytidine diphosphate ProCho, which reacted with diacylglycerol under the influence of choline phosphotransferase to afford phosphatidyl‐propargylcholine (**ProCho‐PC**). The authors were able to visualize **ProCho‐PC** in cells after CuAAC reaction with labeled azide. Lipid analysis of labeled cells showed significant incorporation of propargyl‐Cho into all classes of PC. It was also shown that the fatty acid composition of **ProCho‐PC**‐labeled phospholipids was similar to that of native PC. Cairo et al. [97] and Salic et al. [98] also used 1‐azidoethyl‐choline (AECho) in the same manner to generate in cellulo **AE‐PC** to perform lipid dynamics (lateral mobility) measurements by fluorescence photobleaching recovery and sensitive two‐color imaging of choline phospholipids to get similar results. Bumpus and Baskin [99] used a chemoenzymatic strategy to produce in cellulo PA probes with phospholipase D (PLD) and various alkynols. This enzyme is known to produce PA from PC and make transphosphatidylation in the presence of alcohols. The authors named their methodology as imaging phospholipase D activity with clickable alcohols via transphosphatidylation (IMPACT) [100]. On the one hand, the authors found that the generated PA probes were able to afford the spatial intracellular requirements for PA synthesis in cell signaling. On the other hand, the PA probes were not able to fully recapitulate the biological activities of the native PA. Indeed, the probes allowed only a partial matching with Spo20p, a known interactant of PA (Figure 9).

In 2015, Guo and co‐workers [101] pursued the labeling of GPI‐APs using inositol derivatives. It involved designing and synthesizing clickable *myo*‐inositol substrates by using the nucleophilicity of alcohols to attach azidoethyl tags at the 3‐, 4‐, and 5‐positions, avoiding the sites required for GPI biosynthesis. However, the synthesis of the substrate analogs is challenging due to the need for enantioselective desymmetrization of *myo*‐inositol. Moreover, the free probes were ineffective. To overcome this problem, esterified probes were developed, which exhibited improved membrane permeability and incorporation by cells. Partially acylated probes, such as **4AP‐PI**, proved effective for GPI labeling, confirmed by fluorescence imaging, flow cytometry, and inhibition studies using per‐acetylated *myo*‐inositol. Following this strategy, Best et al. [102] developed **2AP‐PI** to avoid modifying 3–, 4‐, and 5‐positions; these probes may be phosphorylated in cellulo to produce the seven core PIP_n_ isomers. Fluorescence imaging of labeled PI products in cells supported by MS studies showed that tagged PIP probes were detected. They also performed fluorescence‐based thin‐layer chromatography (TLC) experiments in which the dose‐dependent appearance of new spots correlated with commercial PI and PIP_n_ standards.

The second option is to replace the hydroxy group with azide that is frequently used to substitute hydroxy groups, because they are similar in size and dipole moment and replacing a hydroxy group with azide is not likely to have a major impact on the bioavailability and biological behaviors or properties of the molecules. Meanwhile, the azido group facilitates convenient and biocompatible click chemistry for further conjugation. Accordingly, Swarts, Jackson, and co‐workers [103] designed and synthesized a series of azide‐labeled inositol derivatives including **5A‐PI** to explore inositol‐containing glycans, such as phosphatidylinositol mannosides (PIMs), lipoarabinomannans (LAMs), and lipomannans (LMs) in Mycobacteria. Their studies reveal that **5A‐PI** can be selectively incorporated into early PIMs, LAMs, and LMs in *M* *ycobacterium* *smegmatis*, which become visible after click‐based conjugation with fluorophores, and that prolonged exposure of cells to **5A‐PI** created a selective pressure that enabled the isolation of suppressor mutants, ultimately leading to the identification of a novel ABC transporter involved in inositol uptake, thereby linking probe development to insights into inositol uptake, biosynthesis, transport, and dynamics.

Metabolic engineering of cell‐surface GPIs and GPI‐APs using tagged GPI precursors, together with click reactions to attach molecular labels and modern analytical technologies, has wide applications and enables the investigation of many biological problems. For example, metabolic GPI engineering is combined with DNA hybridization chain reaction‐based signal amplification to create a method for sensitive detection and analysis of low‐abundance GPI‐APs on the surface of live cells [104]. Recently, the Guo group also combined metabolic engineering with Transwell coculture to directly analyze intercellular trafficking of global GPI‐APs, demonstrating that GPI‐AP shedding and transfer are GPI‐ and cell type‐dependent and are mechanistically linked to endocytosis, extracellular vesicles, and membrane lipid organization [105].

Last but not least, in 2023, Best et al. [106] demonstrated that N‐L‐SerN_3_ and C‐L‐SerN_3_ were incorporated into the enzymatic pathways of cells yielding **N‐** **L**‐**SerN**_**3**_ **‐PS** and **C‐** **L** **‐SerN**_**3**_ **‐PS** as supported by mass spectrometry studies, thin‐layer chromatography analysis, and derivatization with fluorescent reporters via click chemistry to facilitate imaging in yeast cells. However, treatment with C‐L‐SerN_3_ did not yield detectable mass peaks for labeled PE and PC at retention times matching those of standards. These results are consistent with the hypothesis that conversion of labeled PS to PE via Psd1 or Psd2 is disfavored, given that the carboxyl functionality of C‐L‐SerN_3_ is altered to an amide. Nevertheless, due to its higher metabolic stability, this probe was used by Reynolds et al. [107] to evaluate inhibitors of the *Candida albicans* phosphatidylserine synthase.

## 4 Bioconjugation Using the Glycerol Backbone

**FIGURE 10:**
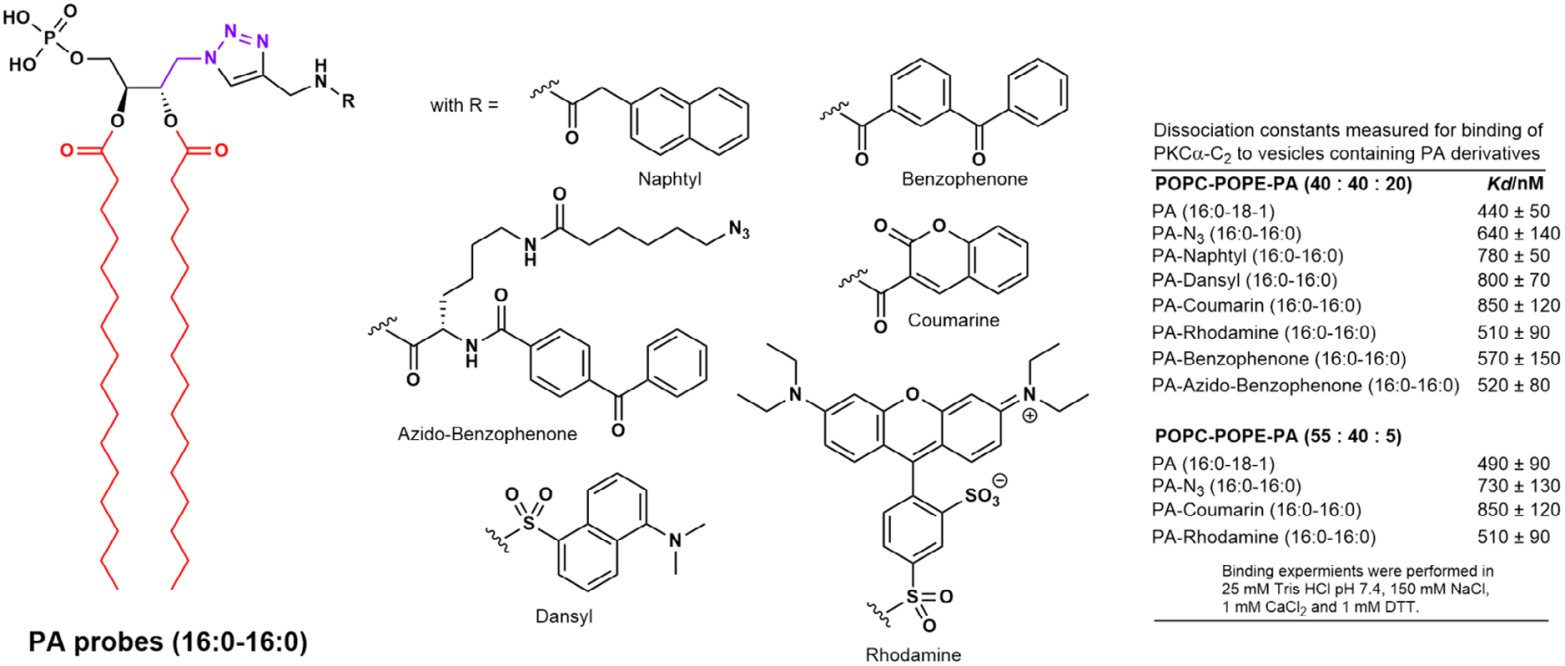
Structures of various PA probes synthesized by Best et al. **PA‐Naphtyl (16:0‐16:0)**, **PA‐Dansyl (16:0‐16:0)**, **PA‐Coumarin (16:0‐16:0)**, **PA‐Rhodamine (16:0‐16:0)**, **PA‐Benzophenone (16:0‐16:0)**, and **PA‐Azido‐Benzophenone (16:0‐16:0)**, as well as their dissociation constants for binding with PCα‐C2.

Using the glycerol skeleton for GPL bioconjugation has long been neglected. The most obvious reason is the difficulty of such an approach, because it involves much greater synthetic challenge than modifying the fatty acyl chain. However, it is well established [108] that the predominant interactions between lipids and proteins occur through the phosphate headgroups and secondarily through interactions with the fatty acyl chains, leaving the glycerol backbone out of the picture. This indifference of proteins toward the latter therefore makes it a prime location for performing bioconjugation while minimizing the risk of interfering with the biological properties of native GPLs. In 2008, Best et al. [109] were the first to modify the glycerol backbone by adding an azidomethyl moiety, allowing CuAAC ligation on diacylglycerol using PCL or fluorescent molecules. One year later, they modified their synthetic pathway to apply this new bioconjugation approach to the first PA‐N_3_ (16:0–16:0) and the respective PCL and fluorescent PA (Figure 10) [110]. They demonstrated the relevance of this strategy by measuring the ability of various synthetic PAs to incorporate into vesicles and interact with the binding domain C2 of protein PKCa (Figure 10). The results indicate clearly that the synthetic PA yielded only slightly weaker affinities—less than two folds—than unmodified PA (16:0–18:1). Moreover, the presence of the azide group allows to graft efficiently by click chemistry a diversity of biological tools such as fluorescent or photocross‐reactive linker moieties. The only limitation, apart from the fact that it only concerns PA, is that the authors only describe access to synthetic PAs having two identical fatty acyl chains. This therefore denies access to the wide diversity of PA, while dozens of PAs are known in mammals [111].

**FIGURE 11:**
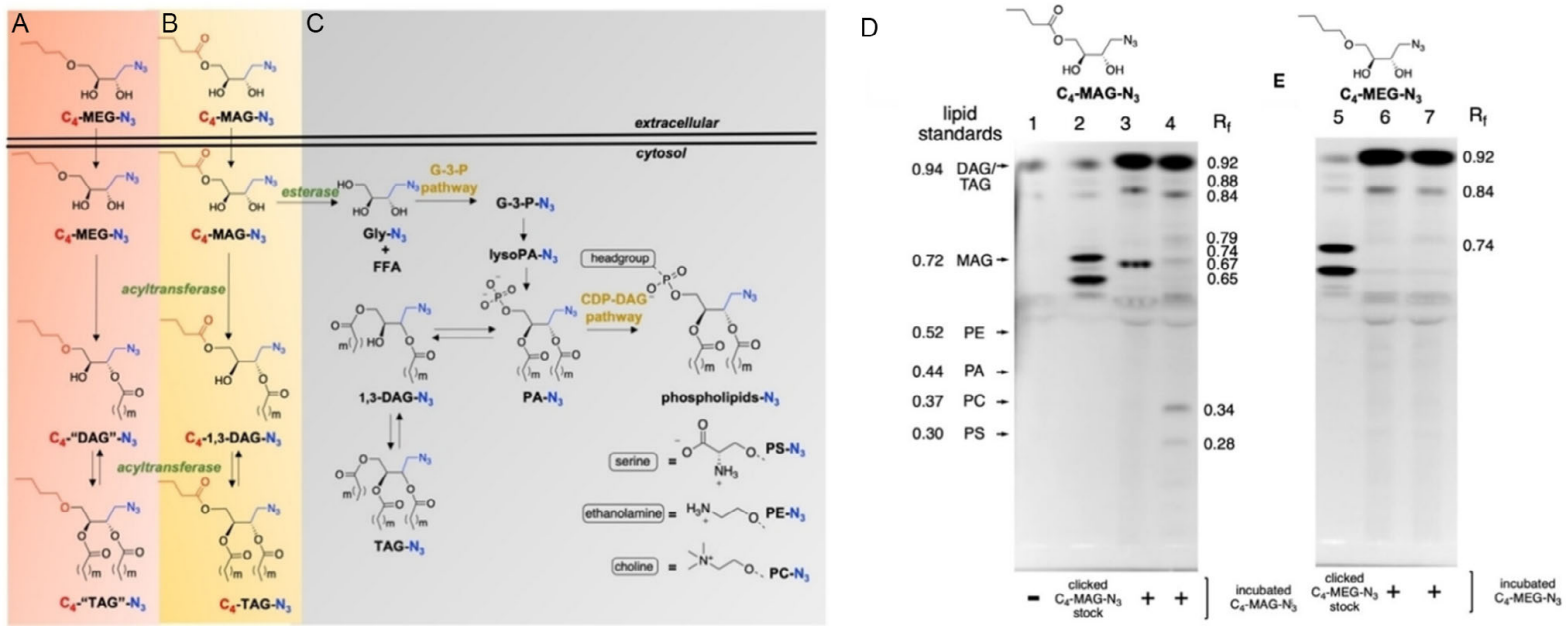
(A–C) Simplified representation of lipid biosynthetic transformations in *S. cerevisiae* indicating potential glycerolipid products from **C**_**4**_‐**MEG‐N**_**3**_ or **C**_**4**_‐**MAG‐N**_**3**_ probes. Synthetically added tails are in red, biosynthetically added fatty acyl tails of varying lengths (m) are in black, and the methylene azide tag is in blue. (A) Acyltransferases can convert **C**_**4**_‐**MEG‐N**_**3**_ into DAG and TAG. (B) Labeled nonpolar lipids can be produced from **C**_**4**_‐**MAG‐N**_**3**_ by acyltransferase. (C) From **C**_**4**_‐**MAG‐N**_**3**_ and esterase, glycerol‐N_3_ can be produced; through the cytidine diphosphate diacylglycerol (CDP‐DAG) pathway, labeled phospholipids can be produced. (D, E) TLC image showing C_4_‐MAG‐N_3_ labels neutral lipids and phospholipids, while **C**_**4**_ **‐MEG‐N**_**3**_ labels only neutral lipids. Lipids were extracted and subjected to CuAAC reaction with ethynylnaphthalimide “naphth.” Clicked extracts were loaded onto a TLC plate for lipid separation by elution with chloroform/methanol/water/acetic acid and then cyclohexane/ethyl acetate, and finally analyzed with fluorescence imaging. (D) Lane 1 contained no probe control lipid extracts. Lane 2 was loaded with a stock of **C**_**4**_‐**MAG‐N**_**3**_ clicked with naphth. Lanes 3 and 4 were naphth‐clicked lipid extracts from yeast incubated with **C**_**4**_‐**MAG‐N**_**3**_. Lane 3 contained lipids isolated from 24‐h incubation, while lane 4 contained lipids obtained from 12‐h incubation. The *R*_f_ values and positions of lipid standards for DAG (16:0—16:0), MAG (18:1), and different phospholipids are indicated with arrows on the left side of the figure (viewed after primulin staining). *R*_f_ values for new spots are shown on the right side of each image. (E) TLC results representing click‐derived fluorescence from lipid extracts when *S. cerevisiae* cells were incubated with 1.0 mM **C**_**4**_‐**MEG‐N**_**3**_. Lane 5 was loaded with a stock of **C**_**4**_‐**MEG‐N**_**3**_ that was clicked with naphth. Lanes 6 and 7 contained lipids from 24 and 12‐h incubation, respectively. Reproduced with permission from ref. [112]. Copyright 2023, Wiley‐VCH GmbH.

A decade later Best et al. [112] incubated yeast (*Saccharomyces cerevisiae* TRY 181) with **C**_**4**_‐**MEG‐N**_**3**_ and **C**_**4**_ **‐MAG‐** **N**_**3**_ that use the same azidomethyl modification of the glycerol core (Figure 11A–C). They found that the **C**_**4**_ **‐MAG‐N**_**3**_ probe was able to successfully infiltrate the lipid metabolism of yeasts and result in fluorescence at the plasma membrane. Labeled lipid classes were identified using TLC (Figure 11D–E), and further characterization down to the lipid species was performed using LCMS. It is shown that probe **C**_**4**_ **‐MAG‐N**_**3**_ is able to be metabolized, resulting in the in cellulo production of **PE‐N**_**3**_, **PC‐N**_**3**_ and **PS‐N**_**3**_.

**FIGURE 12:**
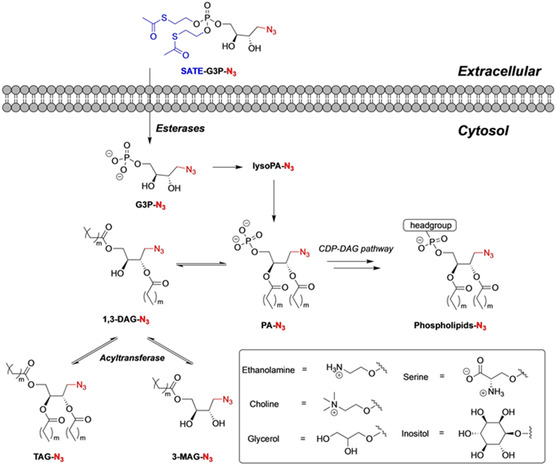
A simplified representation of biosynthetic transformations in *S. cerevisiae* to produce potential labeled glycerolipids from **SATE‐G3P‐N**_**3**_ probe. Upon cell entry, esterases can remove the SATE group, resulting in **G3P‐N**_**3**_ intermediate, which will be acylated to produce **PA‐N**_**3**_. **PA‐N**_**3**_ acts as the central lipid to produce downstream phospholipids via CDP‐DAG pathway, such as PE, PS, PG, PI, and PC. Alternatively, neutral lipids can be obtained via dephosphorylation by phosphatases. The tails can be added/removed by acyltransferases, resulting in DAG, TAG, or MAG lipids. Reproduced with permission from ref. [113]. Copyright 2024, Wiley‐VCH GmbH.

Following these studies, the authors used **SATE‐G3P‐N**_**3**_ [113] with the phosphate moiety caged by S‐acetylthioethyl (SATE) group to incubate with yeast cells. This strategy allows for initial caging of the negatively charged phosphate moiety as a neutral phosphotriester, which upon cell entry undergoes an immolative process driven by intracellular esterase to unveil the native phosphate. The resulting glycerol phosphate‐N_3_ was metabolized to produce various GPL‐N_3_ as demonstrated by the fluorescence microscopy, MS, and TLC analyses performed (Figure 12).

**FIGURE 13:**
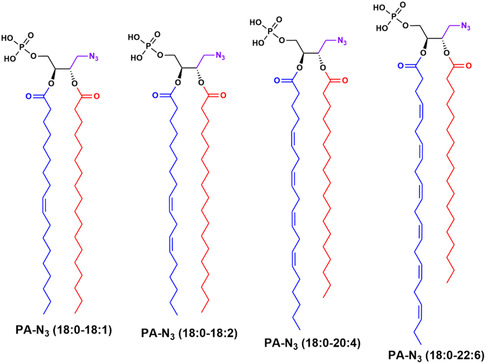
Structures of the **PA‐N**_**3**_ **(18:0–18:1)**, **PA‐N**_**3**_ **(18:0–18:2)**, **PA‐N**_**3**_ **(18:0–20:4)**, **PA‐N**_**3**_ **(18:0–22:6)**.

Both **C**_**4**_ **‐MAG‐N**_**3**_ and **SATE‐G3P‐N**_**3**_ demonstrate that the presence of an azidomethyl moiety on the glycerol core do not prevent them from being metabolized by various enzymes into GPL‐N_3_, further suggesting that the glycerol backbone is a perfect site for the bioconjugations of GPLs. However, if these works demonstrate the ability of Gly‐N_3_ and **G3P‐N**_**3**_ for incorporation into the various enzymatic pathway transforming GPLs, this in cellulo production of GPL‐N_3_ methodology does not allow the controlled biosynthesis of well‐defined GPL probes (including fatty acid chain composition and the nature of the phosphate polar head) and, furthermore, the quantities generated and the complex mixture of resulting GPL‐N_3_ complicate potential future uses. To circumvent these limitations, Balieu et al. [114] described a synthetic method leading to **PA‐N_3_** probes of any fatty acyl chain compositions (Figure 13), which is a breakthrough in the design of GPL‐N_3_ probes.

**FIGURE 14:**
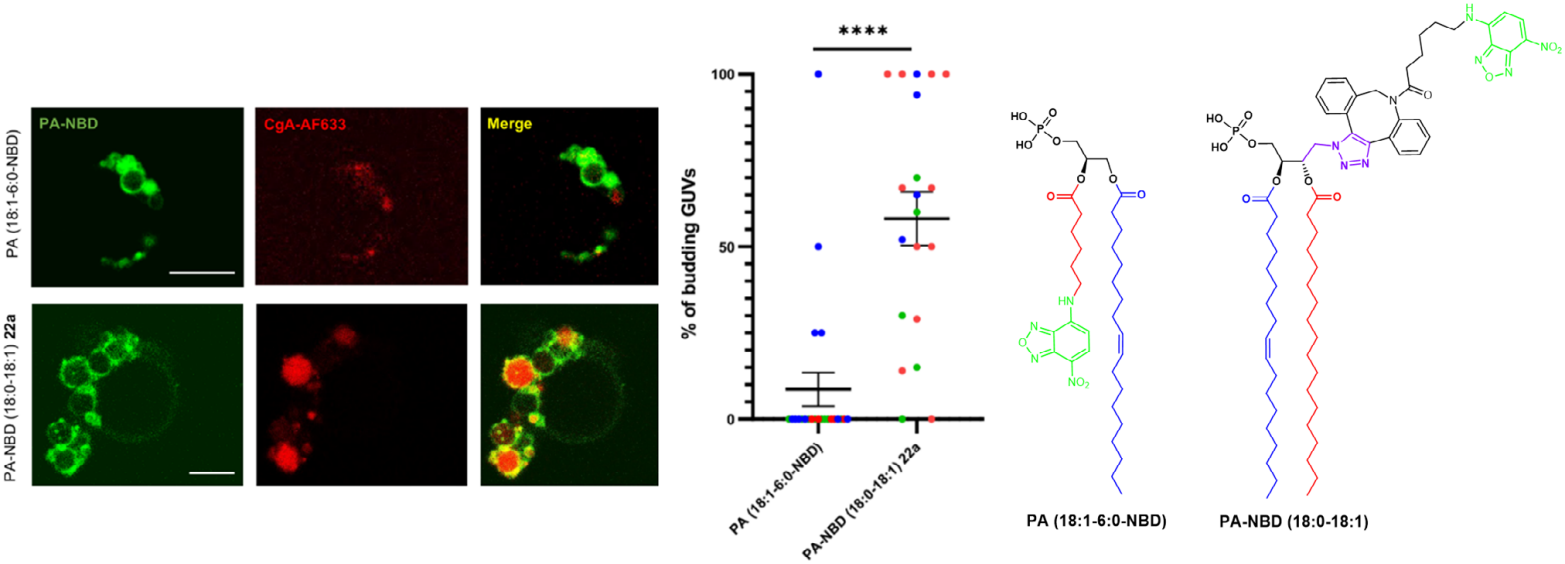
Liposome‐based evaluation of synthetic fluorescent PA‐NBD analogs. Representative images of GUVs composed of 96% DOPC and 4% **PA (18:1–6:0‐NBD)** or **PA‐NBD (18:0–18:1)**, in the presence of CgA‐AF633. Scale bar, 10 μm. Quantification of budding: each point represents the result of GUV budding analysis from three independent experiments (20 and 22 images for each type of PA‐NBD‐containing GUVs, respectively). Data points are colored coded (red, blue, or green) according to experimental repeats. ^∗∗∗∗^: *p <* 0.0001, Mann–Whitney test.

The transformation of **PA‐N**_**3**_ into fluorescent and photoreactive PA probes was performed via SPAAC with various DIBAC partners. These probes were first tested for their abilities to participate in membrane remodeling using giant unilamellar vesicles (GUVs) carrying labeled chromogranin A (CgA), a glycoprotein known to induce membrane remodeling in the presence of PA [115]. GUVs containing **PA‐NBD (18:0–18:1)** exhibited significant budding events in agreement with what was previously observed and the fluorescence microscopy images showed colocalization of both partners (Figure 14). In contrast, CgA‐AF633 failed to induce the budding of GUVs containing commercially available **PA (18:1–6:0‐NBD)** bearing a modification on the fatty acyl chain. Quantitative analysis confirmed that significantly more budding were observed with **PA‐NBD (18:0–18:1)** than with **PA (18:1–6:0‐NBD)**, demonstrating that CgA interacts effectively with **PA‐NBD (18:0–18:1)**. This interaction is functional since the new probe can mimic the cellular properties of native PA (18:0–18:1) in Golgi and secretory granule membranes, whereas **PA (18:1–6:0‐NBD)** failed to replicate those effects.

The biocompatibility and the membrane incorporation of synthetic mono‐ and poly‐unsaturated PA analogs in neurosecretory cells were also evaluated. Primary chromaffin cell live‐imaging revealed that **PA‐ATTO 647N (18:0‐18:1)** and **PA‐ATTO 647N (18:0–22:6)** analogs rapidly (in dozens of seconds) inserted into cellular membranes and accessed intracellular compartments without visible alteration, and this was confirmed utilizing the PA sensor Spo20p‐GFP. The latter was recruited to the plasma membrane within minutes of incubation of **PA‐N**_**3**_ **(18:0–18:1)**, **PA‐N**_**3**_ **(18:0–22:6)**, **PA‐DDA1 (18:0–18:1)**, or **PA‐DDA1 (18:0–22:6)**. These data confirm the integrity and stability of synthetic PA in cellulo, as well as their ability to interact with a known PA‐binding protein. Based on these results, Montero‐Hadjadje et al. [116] performed the first FLIM‐FRET study to monitor protein‐lipid interactions in a dynamic context with CgA‐mKate2 and **PA‐ATTO 647N (18:0–18:1)** in COS‐7 cells. They were able to measure marked decrease in CgA‐mKate2 lifetime following **PA‐ATTO647N (18:0–18:1)** addition (from 2.47 ± 0.19 to 2.08 ± 0.09 ns), indicating robust FRET and thus direct proximity between CgA and PA, meaning a very probable interaction between the protein and the lipid. In parallel, Balieu et al. [114] performed functional validation of synthetic PA through rescue experiments measuring neurosecretion *via* exocytosis labeling. Inhibition of DβH staining caused by the PLD1 inhibitor CAY93 was rescued by synthetic **PA (18:0–18:1)**, whereas synthetic **PA (18:0–22:6)** failed to restore DβH labeling, consistent with results obtained with natural PA. Similarly, the modified **PA (16:0‐6:0‐NBD)** did not rescue the inhibition. Electrochemical recordings showed that CAY93 reduced the number of exocytotic events, an effect not reversed by poly‐unsaturated PA species. However, CAY93 also slowed fusion events and prolonged fusion pore duration, effects that were successfully rescued by PA (18:0‐22:6) and **PA‐DDA1 (18:0–22:6)**. These results indicate that the synthetic PA analogs reproduce the specific functions of natural mono‐ and poly‐unsaturated PA during neuroendocrine secretion, unlike **PA (18:0–6:0‐NBD)**, suggesting that glycerol backbone modifications do not alter their cellular functions.

**FIGURE 15:**
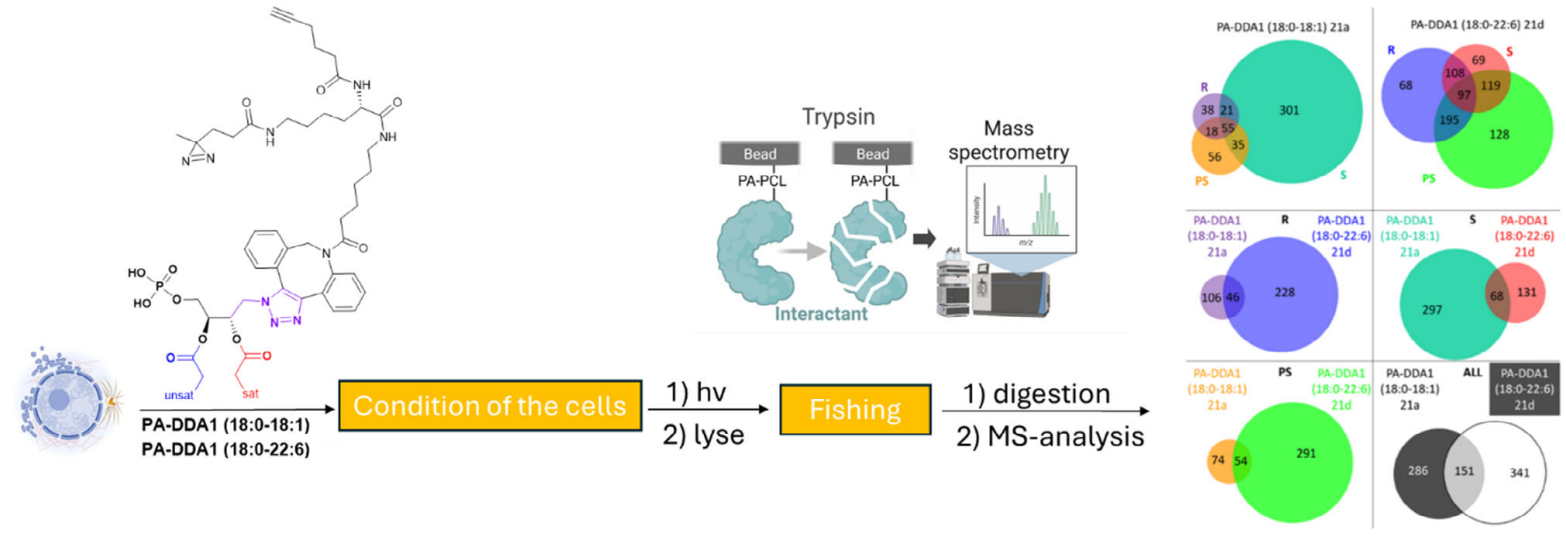
PA interactome in neurosecretory cells. Distribution of proteins identified in the fishing experiments performed with bovine chromaffin cells for **PA‐DDA1 (18:0–18:1)** and **PA‐DDA1 (18:0–22:6)** in resting condition (R), stimulated condition (S), or resting condition poststimulation (PS).

Balieu et al. [114] and Vitale et al. [117] also performed the characterization of PA interactome in neurosecretory cells using synthetic **PA‐DDA1**. Chromaffin cells were incubated with **PA‐DDA1 (18:0–18:1)** or **PA‐DDA1 (18:0–22:6)**, illuminated to cross‐link PA interactants under resting, stimulated, or poststimulation conditions, and purified using beads bearing azide *via* CuAAC with the alkyne moiety of DDA1. Interacting proteins were then identified by mass spectrometry. After removing the background polypeptides detected without illumination, 778 potential PA partners were identified, including previously reported PA interactants, validating the approach. Numerous new candidates were also discovered, with 286 specifics to PA (18:0–18:1), 341 to PA (18:0–22:6), and 151 shared between both species (Figure 15). Some interactants were condition‐specific, highlighting the dynamic nature of PA‐protein interactions during neurosecretion.

To reach other families of GPL‐N_3_, Balieu et al. [118] have extended their synthetic methodology to **PA‐N**_**3**_. Thus, they were able to obtain **PE‐N**_**3**_ **(16:0–18:1)**, **PE‐N**_**3**_ **(18:0–18:1)**, **PC‐N**_**3**_ **(16:0–18:1)**, and **PS‐N**_**3**_ **(16:0–18:1)** thanks to a convergent approach for previously synthesized **PA‐N**_**3**_ by using the pyrophosphate chemistry and varied alcohols. This innovation also provides powerful tools for selective cancer cell labeling with **PE‐N**_**3**_ **(18:0–18:1)** by exploiting the differences in composition of the membrane leaflets between normal and cancer cells, thanks to in cellulo SPAAC using various fluorescent partners. Finally, SPAAC with a dinitrophenyl‐DBCO derivative demonstrates the potential of using **PE‐N**_**3**_ **(18:0–18:1)** to recruit endogenous anti‐DNP antibodies to the cell surface, with in mind the implementation to targeted immunotherapy.

## 5 Summary and Outlook

Synthetic lipid biology focuses on designing and manipulating lipids, membranes, and associated proteins to control their production, organization, dynamics, and interactions in biological systems for gaining knowledge in this field of research. Bioconjugation of GPL initially rooted in multistep synthetic chemistry, which enables precise molecular modifications, but the field is in extension with the use of the metabolic pathway to produce lipidic probes allowing easier production and spatiotemporal fidelity with the native lipids in living cells, thanks to bioconjugate substrates. Thus, future research in this field will build on these two approaches, which are not in competition but rather complementary. Indeed, the information obtained more rapidly through the metabolomic approach, which requires less organic synthesis, will need to be corroborated by the use of probes with well‐defined structures, since the metabolomic approach generally does not produce a single species but rather a family of phospholipids.

Despite these advances, major challenges remain due to the inherent complexity of GPL, including their structural similarity, rapid transport, and metabolism as well as their transient interactions with other biomolecules, and due to the limited accurate tools available for their detection and modeling. Looking forward, progress in probe design, computational biology, artificial intelligence, and interdisciplinary collaboration is expected to help overcome these limitations and improve the understanding of lipid biology, driving applications in medicine and biotechnology.

## Author Contributions

**Brice Beauvais**: writing – review and editing, writing – original draft. **Rajendra Rohokale**: writing – review and editing, writing – original draft. **Sevin Mamputu**: writing – original draft. **Ion Lupu**: writing – original draft. **Zhongwu Guo**: writing – review and editing, resources, supervision, writing – original draft, funding acquisition. **Sébastien Balieu**: conceptualization, writing – review and editing, writing – original draft, funding acquisition, resouces, supervision.

## Funding

This work was supported by the Conseil National de la Recherche Scientifique, European Regional Development Fund, Agence Nationale de la Recherche (ANR‐11‐LABX‐0029, ANR‐18‐EURE‐0020XL CHEM, ANR‐25‐CE44‐1554 TACLE), NIH/NIGMS (R35 GM131686), Fulbright (U.S. Department of State and the Franco‐American Commission), and Carnot Institute I2C.

## Conflicts of Interest

The authors declare no conflicts of interest.

## Data Availability

The data that support the findings of this study are available from the corresponding author upon reasonable request.
